# Carbon Source Affects Synthesis, Structures, and Activities of Mycelial Polysaccharides from Medicinal Fungus *Inonotus obliquus*

**DOI:** 10.4014/jmb.2102.02006

**Published:** 2021-04-21

**Authors:** Huihui He, Yingying Li, Mingyue Fang, Tiantian Li, Yunxiang Liang, Yuxia Mei

**Affiliations:** State Key Laboratory of Agricultural Microbiology, College of Life Science and Technology, Huazhong Agricultural University, Wuhan 430070, P.R. China

**Keywords:** *Inonotus obliquus*, carbon source, liquid fermentation, polysaccharide synthesis, structural properties, activities

## Abstract

The effects of various carbon sources on mycelial growth and polysaccharide synthesis of the medicinal fungus *Inonotus obliquus* in liquid fermentation were investigated. After 12-d fermentation, mycelial biomass, polysaccharide yield, and polysaccharide content were significantly higher in Glc+Lac group (glucose and lactose used as combined carbon source) than in other groups. Crude polysaccharides (CIOPs) and the derivative neutral polysaccharides (NIOPs) were obtained from mycelia fermented using Glc, fructose (Fru), Lac, or Glc+Lac as carbon source. Molecular weights of four NIOPs (termed as NIOPG, NIOPF, NIOPL, and NIOPGL) were respectively 780.90, 1105.00, 25.32, and 10.28 kDa. Monosaccharide composition analyses revealed that NIOPs were composed of Glc, Man, and Gal at different molar ratios. The NIOPs were classified as α-type heteropolysaccharides with 1→2, 1→3, 1→4, 1→6 linkages in differing proportions. In in vitro cell proliferation assays, viability of RAW264.7 macrophages was more strongly enhanced by NIOPL or NIOPGL than by NIOPG or NIOPF, and proliferation of HeLa or S180 tumor cells was more strongly inhibited by NIOPG or NIOPGL than by NIOPF or NIOPL, indicating that immune-enhancing and anti-tumor activities of NIOPs were substantially affected by carbon source. qRT-PCR analysis revealed that expression levels of phosphoglucose isomerase (PGI) and UDP-Glc 4-epimerase (UGE), two key genes involved in polysaccharide synthesis, varied depending on carbon source. Our findings, taken together, clearly demonstrate that carbon source plays an essential role in determining structure and activities of *I. obliquus* polysaccharides by regulating expression of key genes in polysaccharide biosynthetic pathway.

## Introduction

Medicinal fungi have received considerable research attention because of the various bioactivities displayed by their metabolites. These metabolites typically have complex and novel structures, and are an important source of precursors in drug discovery and functional foods. *Inonotus obliquus*, commonly known as 'chaga,' is a black parasitic fungus (division Basidiomycota, family *Hymenochaetaceae*) [1] that has been widely used in Russia and Eastern Europe since at least the 16th century as a treatment in folk medicine for cancer, cardiopathy, hepatopathy, gastropathy, and diabetes [2]. Numerous studies have focused on production and pharmacological activities of active metabolites of *I. obliquus*, which include triterpenoids, polyphenols, fuscoporine, and polysaccharides. In particular, *I. obliquus* polysaccharides (IOPs) have been found to display notable immunostimulatory [3], anti-cancer [4], anti-oxidant [5], anti-fatigue [6], and hypoglycaemic [7] activities.

The natural distribution of *I. obliquus* is mainly between 45° and 50° north latitude, which includes Heilongjiang and Jilin provinces of China, Siberia, northern Europe, and Japan. Because of the harsh environments in which *I. obliquus* grows, wild resources are limited and extremely expensive to obtain. Artificial culture techniques have been developed in response to consumer demand; these include wooden culture, solid-state fermentation, and liquid fermentation. Liquid fermentation, because of its short fermentation period, relatively low cost, and high metabolite production, is a useful method for producing large quantities of *I. obliquus* metabolites, particularly IOPs. An increasing number of studies during the past decade have focused on optimization of liquid fermentation parameters for enhancing production of *I. obliquus* mycelial biomass and active metabolite yield. Wei *et al*. developed a glucose fed-batch integrated dissolved oxygen (DO) control strategy for *I. obliquus* liquid fermentation that resulted in significant enhancement of biomass and production of polysaccharides, triterpenoids, and inotodiol [1]. In a forced air injection system, a nitrogen:oxygen ratio of 50:50 gave the best results in terms of biomass and contents of triterpenes and betulinic acid [8]. Certain stimulatory agents (VB6, farnesol, Tween 80) also enhanced mycelial biomass and contents of extracellular polysaccharides, triterpenoids, and betulinic acid in *I. obliquus* liquid fermentation [9-11]. These and other studies have demonstrated substantial improvement of *I. obliquus* growth and/or metabolite production by a variety of optimization strategies in terms of fermentation medium and/or processing conditions; however, such studies have generally not addressed the structural characterization or bioactivity of metabolites.

We investigated the effects of fermentation medium components on synthesis, structures, and bioactivities of *I. obliquus* mycelial polysaccharides generated by liquid fermentation. Glucose (Glc), fructose (Fru), and lactose (Lac) were used as single or combined carbon sources. Crude polysaccharides and neutral polysaccharides were extracted and purified from mycelia based on various carbon sources. These polysaccharide fractions were subjected to structural characterization and comparative analysis of immune-enhancing and anti-tumor effects. In addition, we evaluated the effects of different carbon sources on expression levels of related key enzymes involved in IOP biosynthetic pathway.

## Materials and Methods

### Materials and Reagents

*I. obliquus* was obtained from the Microbial Genetic Stock Center of Huazhong Agricultural University (Wuhan, China). DEAE cellulose-52, Sephadex G-100, and T-series dextran molecular weight standards (T-10, T-40, T-70, T-500, T-2000) were from Pharmacia (Sweden). D-glucose (Glc), D-mannose (Man), D-arabinose (Ara), D-xylose (Xyl), D-fucose (Fuc), L-rhamnose (Rha), inositol, and erythritol (purity of standards ≥99%) were from Sigma-Aldrich (USA). DMEM, RPMI-1640, trypsin, penicillin-streptomycin, and diethyl pyrocarbonate (DEPC)-treated water were from Gibco (USA). High-Capacity cDNA Reverse Transcription Kit was from Thermo Fisher Scientific (USA). HiPure Fungal RNA Mini Kit was from Megen (China). HiScript II Q RT SuperMix for qPCR Kit was from Vazyme (China). Cell Counting Kit-8 (CCK-8 Kit) was from Beyotime Institute of Biotechnology (China). Concanavalin A (ConA) and camptothecin (CPT) were from Macklin Biochemical Co.(China) Macrophage RAW264.7, human cervical cancer HeLa, and mouse sarcoma S180 cell lines were from American Type Culture Collection (ATCC; USA). RAW264.7 was cultured with 10% FBS and 1% double-strength RPMI-1640, and HeLa and S180 were cultured with 10% FBS and 1% DMEM, for 2 days at 37°C in 5% CO_2_ atmosphere. Other reagents were from Sinopharm Chemical Reagent Co. (China).

### Liquid Fermentation of *I. obliquus* with Various Carbon Sources

*I. obliquus* was inoculated on potato dextrose agar (PDA) slants and cultured for 7 days at 28°C. Mycelia were harvested and inoculated to seed medium, which consisted of (all following concentrations g/l): Glc (20), malt extract (6), peptone (5), yeast extract (3), MgSO_4_•7H_2_O (3), KH_2_PO_4_ (0.4), and K_2_HPO_4_ (0.2). Fermentation medium consisted of yeast extract (10), ZnSO_4_•7H_2_O (0.1), and carbon source (30) at initial pH 5.6. Nine carbon sources were used: Glc, Man, Gal, Xyl, Fru, sucrose (Suc), maltose (Mal), Lac, and starch. For liquid fermentation, mycelium suspension from seed culture (final concentration 10%, v/v) was inoculated into 100 ml fermentation medium, and cultured for 12 days at 28°C with rotation (160 r/min). Mycelia were harvested, dried to constant weight at 60°C, ground to powder, and stored at 4°C. Polysaccharide yield and content were calculated according to the following formulas:

Polysaccharide content (g/l) = Weight of polysaccharide (g)/Volume of the fermented broth (L)

Polysaccharide yield (%) = [ Weight of polysaccharide (g)/Mycelial weight (g)] × 100

### Extraction and Purification of *I. obliquus* Polysaccharides

Mycelial powder was mixed with hot water (solid/ liquid ratio 1:40, v/v) at 90°C for 3 h, deproteinized using 1/5 volume of Sevag solvent (chloroform/ n-butanol 4:1, v/v), and centrifuged (8 000 ×g) for 5 min at room temperature. The top supernatant was collected, precipitated with 4 volumes of ethanol solution, and centrifuged as above. Then, crude *I. obliquus* polysaccharides (termed CIOPs) were obtained. The CIOP solution was loaded on a DEAE-52 column (1.5 × 20 cm) pre-equilibrated with distilled water, and eluted with double-distilled water and NaCl solution (0.5 mol/l) at flow rate 1 ml/min. Eluates were collected, and carbohydrate content was measured by phenol-sulfuric acid method [12]. Material was further purified by gel filtration chromatography using a Sephadex G-100 column (1.5 × 60 cm), and then eluted with double-distilled water to yield purified polysaccharide fractions. These polysaccharide fractions were neutral polysaccharides and thus termed as NIOPs. Carbohydrate and protein contents of CIOPs and NIOPs were assayed respectively by phenol-sulfuric acid method and Bradford method [13].

### Molecular Weight (MW) and Monosaccharide Composition of NIOPs

MW of NIOPs was determined using a calibration curve generated by plotting weights of T-series dextran standards against retention time. Monosaccharide compositions of NIOPs were analyzed by gas chromatography (GC) as described by Wang *et al*. [14] with minor modification. Ten milligrams of each NIOP sample and 2 ml trifluoroacetic acid (TFA) (4 mol/l) were sealed with N2, hydrolyzed at 120°C for 3 h, evaporated at 90°C, and added with methanol to remove excess TFA. Dried hydrolysate (10 mg) was mixed with inositol (5 mg), hydroxylamine hydrochloride (10 mg), and pyridine (0.5 ml), incubated at 90°C for 30 min, cooled to room temperature, added with acetic anhydride (0.5 ml), incubated again at 90°C for 30 min, and centrifuged (8 000 ×g) for 5 min at room temperature. Supernatant was collected and analyzed by GC (model 6890 N; Agilent Technologies) with phenyl methylsiloxane capillary chromatographic column (Hp-5; 30 × 320 × 0.25 μm) and flame ionization detector. Column temperature was maintained at 120°C for 3 min, and 230°C for 4 min. Monosaccharide standards were analyzed by GC procedure as above.

### Fourier Transform Infrared Spectroscopy (FTIR)

Dried NIOP samples (2 mg) were mixed with KBr (200 mg) and ground to thin pellets in an agate mortar under infrared lamp. The thin section was analyzed using a Nicolet Nexus FTIR 470 spectrophotometer (Thermo Scientific Nicolet; USA) over wavelength range 400 to 4,000 cm^-1^ [15].

### Periodate Oxidation and Smith Degradation

Each NIOP sample was subjected to periodate oxidation and Smith degradation by the method of Zhang *et al*.[16]. A twenty-milligram NIOP sample was dissolved with 20 ml NaIO_4_ (15 mM) at 4°C in the dark. Reaction solution was added with ethylene glycol (0.4 ml), and absorbance measured by spectrophotometry at wavelength 223 nm at 24-h intervals. HIO_4_ consumption and formic acid production were assayed by titration with NaOH (10 mM). Periodate product was added with ethylene glycol (2 ml), stirred for 30 min, dialyzed with flowing water for 24 h and then distilled water for 24 h, and concentrated to a volume of 10 ml. The resulting concentrated solution was mixed with 50 mg NaBH4, stirred in the dark at room temperature for 24 h, pH-adjusted to 5.5 with 0.1 M acetic acid, dialyzed, and evaporated. The product was subjected to GC analysis as described above.

### In Vitro Cell Viability Assay

Immune-enhancing activity of IOPs was evaluated using RAW264.7 cells. Log-phase cells were added with RPMI-1640 to concentration 5 × 10^5^/ml. Cell suspensions (100 μl) were mixed with 10 μl CIOPs at concentration 10-500 μg/ml or with NIOPs at concentration 1-100 μg/ml, and incubated on 96-well plates in 5% CO_2_ atmosphere at 37°C. After 48 h, the mixture was added with 10 μl CCK-8 solution, incubated for 3 h, and OD_450_ was measured. Positive control was treated with ConA at concentration 1 μg/ml, and normal control was not treated with any drug. Treatments were performed in triplicate. Cell viability was calculated as:

Cell viability (%) = [OD_450_(sample) - OD_450_(control)] × 100

Anti-tumor activity was evaluated using HeLa and S180 cells. IOP samples (final concentration 500 and 1,000 μg/ml) and HeLa/ S180 cells (5 × 10^4^/ml) were cultured on a 96-well plate for 24 h at 37°C. CPT at concentration 600 μg/ml was used for positive control. Cell viabilities were determined by CCK-8 kit and calculated by the above formula.

### Quantitative Real-Time PCR (qRT-PCR)

Transcription levels of phosphoglucose isomerase (PGI), UDP-Glc 4-epimerase (UGE) and β-actin (reference) genes were analyzed by qRT-PCR. *I. obliquus* mycelial powder (50 mg) was prepared for extraction of RNA using a HiPure Fungal RNA Mini Kit. Total generated RNA was dissolved in diethylpyrocarbonate (DEPC) water and transcribed to cDNA as per the HiScript IIQ RT SuperMix protocol for the qPCR kit. The resulting cDNA was used as template. Primer sequences were (5′→3′): PGI: F: ACCACGCAGGAGACGATCAC, R: TGTTGTCAGCCGAGA TACCA; UGE: F: TCGGGAACGACTATCCGAC, R: AGCCTTAAACCGTCCTGTAC; β-actin: F: CCACGA GACAACATACAACT, R: TACCACCAGACAGCACAAC. The qRT-PCR program was: 95°C for 3 min (stage 1); 40 cycles of 95°C for 10 sec, 60°C for 20 sec (stage 2); and 95°C for 15 sec, 60°C for 60 sec, and 95°C for 15 sec (stage 3). Reactions were performed in triplicate. Relative expression levels of PGI and UGE were normalized and determined by 2^-ΔΔCt^ method [17].

### Statistical Analysis

All experiments were performed in triplicate. Data were expressed as mean ± SD, and differences between means were analyzed using software program SPSS for Windows, V. 20 (SPSS, Inc.; USA). Datasets involving more than two groups were assessed by one-way ANOVA followed by Duncan’s multiple range test, and differences indicated by differing superscript letters were considered statistically significant (*p* < 0.05). Comparisons between means of two groups were analyzed using Student’s t-test, and differences with *p* < 0.05 or *p* < 0.01 were considered significant or highly significant, respectively [18].

## Results

### Effects of Carbon Sources on Mycelial Biomass and Polysaccharide Production of *I. obliquus*

**Fig. 1:**
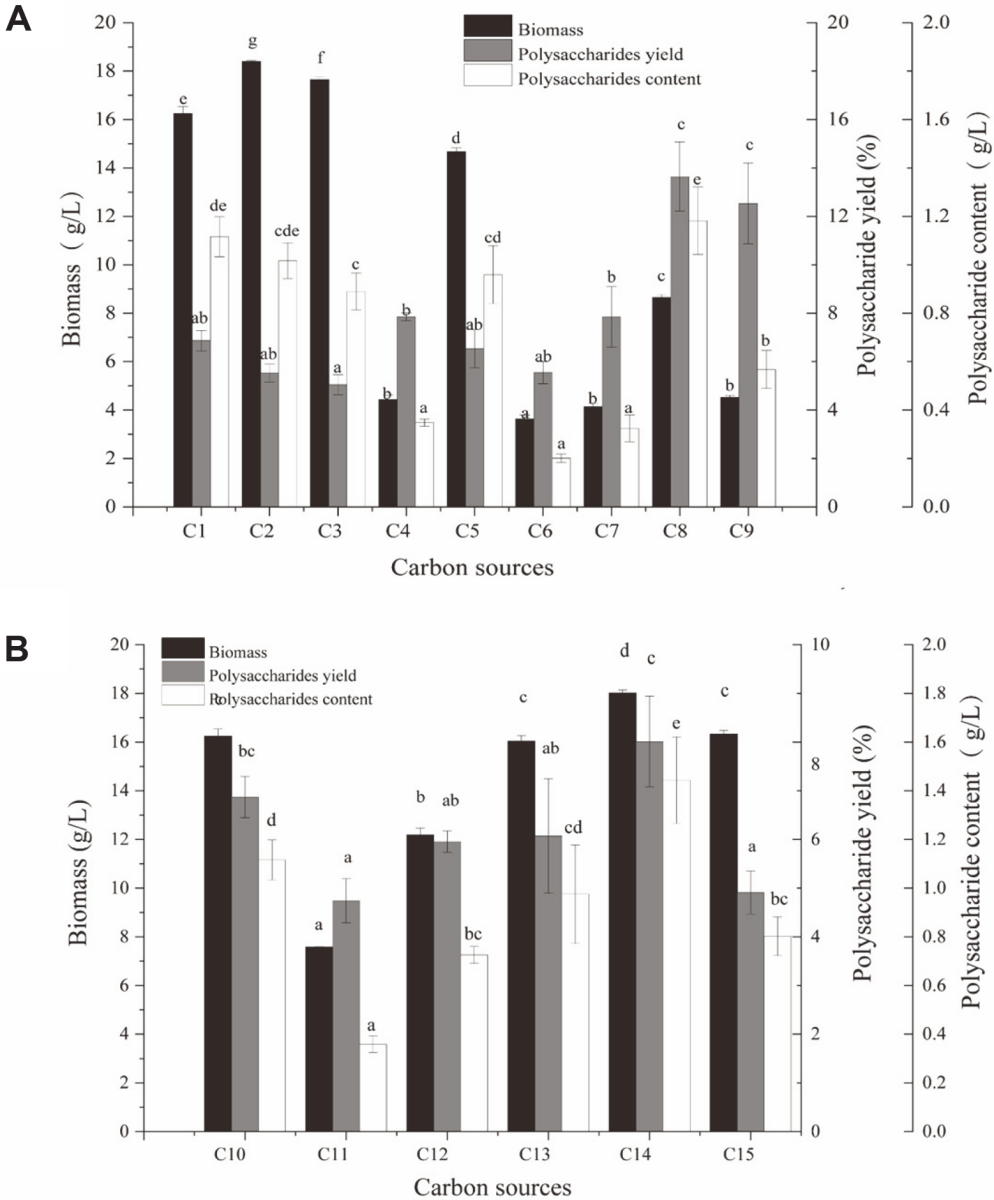
Effects of carbon source on biomass and polysaccharide production of *Inonotus obliquus*. (**A**) Single carbon sources. (**B**) Complex carbon sources. C1-C9: Glc, Fru, Man, Gal, Xyl, Suc, Mal, Lac, and starch; C10-C15: Glc, Glc + Gal, Glc + Suc, Glc + Mal, Glc + Lac, Glc + starch. Differing letters above bars indicate significant (*p* < 0.05) differences according to Duncan’s multiple range test.

*I. obliquus* was cultured on optimized medium, using Glc, Fru, Man, Gal, Xyl, Suc, Mal, Lac or starch as carbon source, and mycelial biomass and polysaccharide content were measured on day 12. Highest biomass values were observed when sole carbon source was Glc, Fru, Man, or Xyl (Fig. 1A). Both biomass and polysaccharide content were high for the Glc group, indicating that Glc is a good carbon source for mycelial growth and polysaccharide production. Although Lac, starch, Mal, and Gal groups had lower biomass values, their polysaccharide yields were higher than those of Glc, Fru, Man, and Xyl groups. We therefore performed experiments with a "mixotrophic" strategy using Glc and a supplementary carbon source in molar ratio 2:1. Mycelial biomass, polysaccharide yield, and polysaccharide content were all significantly higher for the Glc+Lac group (Glc and Lac [2:1] used as combined carbon source) than for other mixotrophic groups (Fig. 1B).

### Isolation and Purification of Polysaccharides from *I. obliquus* Mycelia

**Fig. 2:**
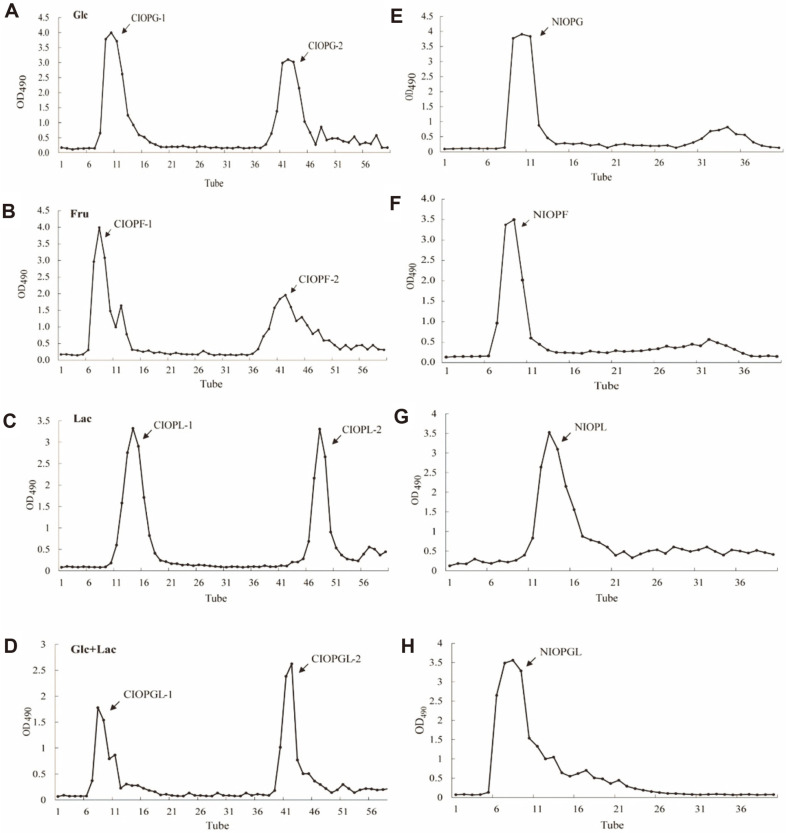
Isolation and purification of crude polysaccharides (A-D) and neutral polysaccharides (E-F) from *I. obliquus* mycelia. Carbon sources used were Glc (A,E), Fru (B,F), Lac (C,G), and Glc+Lac (D,H).

*I. obliquus* was grown for 12 days by liquid fermentation using Glc, Fru, Lac, or Glc+Lac as carbon source, mycelia were harvested, and polysaccharides were isolated by hot water extraction under optimal conditions. Four crude *I. obliquus* polysaccharides (CIOPs) were generated, and termed CIOPG (Glc as carbon source), CIOPF (Fru as carbon source), CIOPL (Lac as carbon source), and CIOPGL (Glc+Lac as carbon source). Each of the above four CIOPs was separated by DEAE-52 column chromatography into two fractions, i.e., CIOPG-1 and -2 (Fig. 2A), CIOPF-1 and -2 (Fig. 2B), CIOPL-1 and -2 (Fig. 2C), and CIOPGL-1 and -2 (Fig. 2D). Each of the above -1 fractions was eluted with water and classified as neutral sugar, and each -2 fraction was eluted with 0.5 M NaCl solution and classified as acid sugar. The four CIOPs differed in regard to contents and ratios of neutral and acid sugars (Figs. 2A-2D).

Because of the abundance of neutral polysaccharides in the four CIOPs, the -1 fractions were further purified by Sephadex G-100 column chromatography. Four purified neutral polysaccharides (NIOPs) were thus generated, and termed NIOPG, NIOPF, NIOPL, and NIOPGL (Figs. 2E-2H). Carbohydrate contents differed notably among the four CIOPs and four NIOPs (Table 1).

### Effects of Carbon Source on Structural Properties of *I. obliquus* Mycelial Polysaccharides

**Fig. 3:**
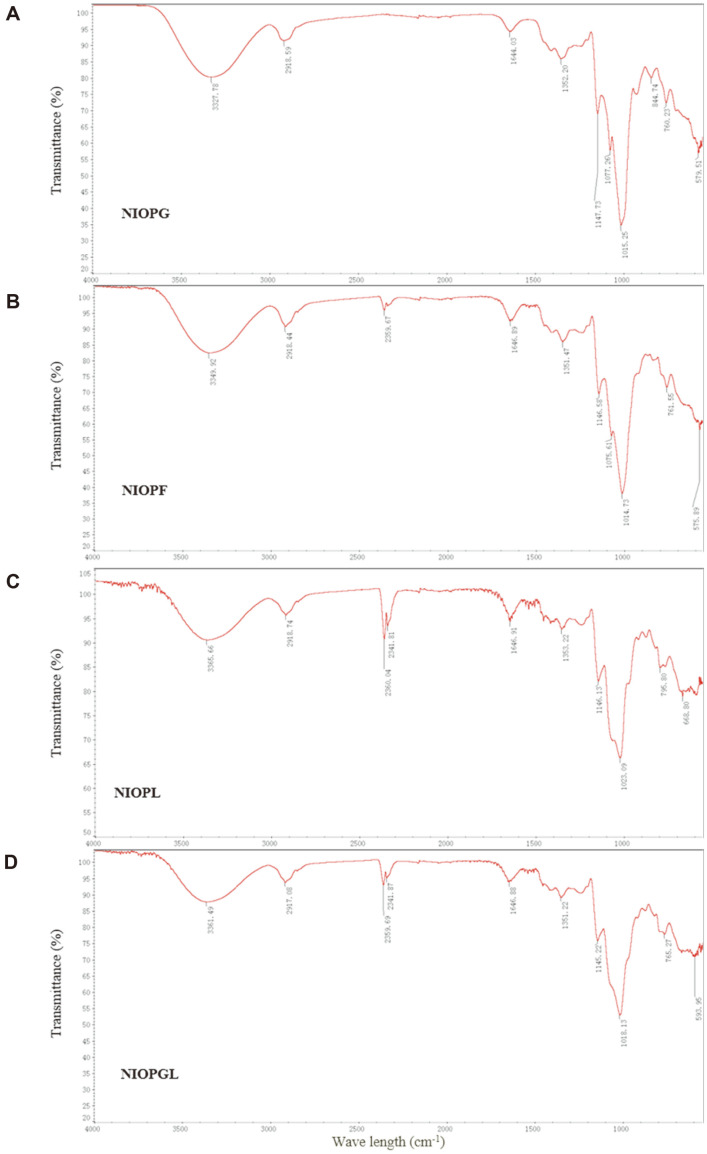
FTIR spectra of NIOPG (A), NIOPF (B), NIOPL (C), and NIOPGL (D).

Carbohydrate content of each of the four NIOPs was >90%, much higher than values for the corresponding CIOPs, which ranged from 18.88% to 33.48% (Table 1). The NIOPs were therefore used for analysis of structural properties. They had typical carbohydrate spectra upon FTIR analysis (Fig. 3). These spectra had similar peaks at 3,327.78-33,615.66 cm^-1^ (O-H stretching), 2,917.08-2,918.74 cm^-1^ (C-H stretching), 1,644.03-1,646.91 cm^-1^ (C=O stretching), 1,351.22-1,353.22 cm^-1^ (asymmetrical C-H bending of CH2 group), and 1,014.73-1,147.73 cm^-1^ (C-O-C) [19]. Absorption peaks at 575.89-593.95 cm^-1^ were attributed to α-glycosidic bond [14]. Three peaks around 1000-1200 cm^-1^ in NIOPG and NIOPF indicated the presence of pyranose (Figs. 3A and 3B). In contrast, NIOPL and NIOPGL had two peaks in that range, suggesting the presence of furanose (Figs. 3C and 3D).

**Fig. 4:**
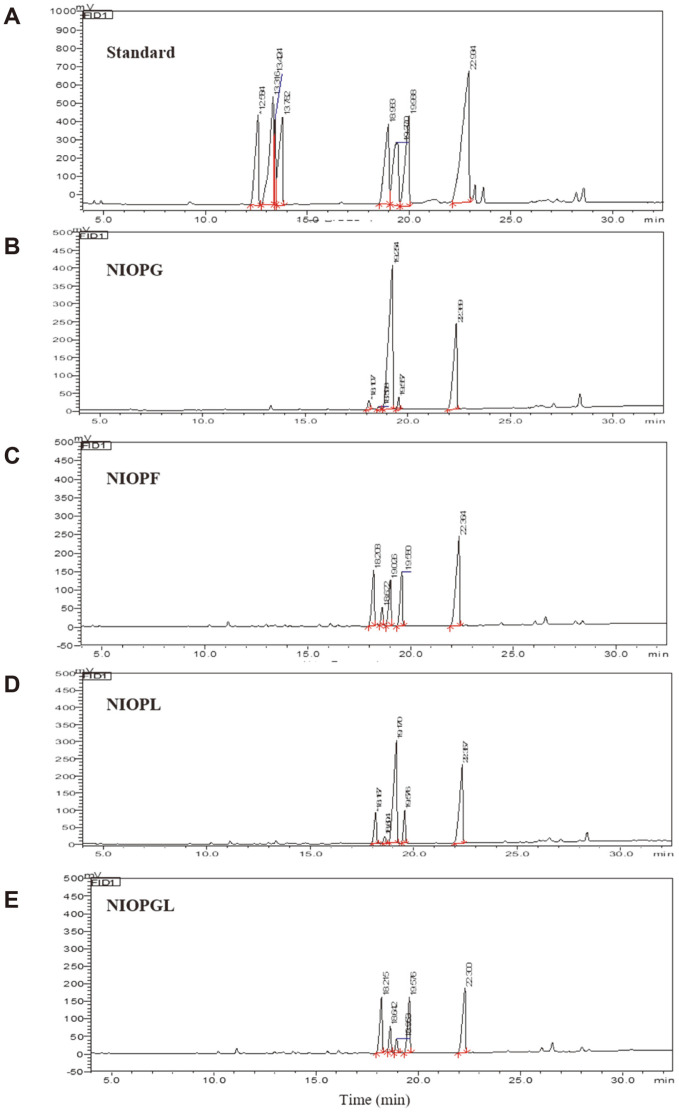
GC-MS analysis of standard monosaccharide mixture (A) and hydrolysis products of NIOPG (B), NIOPF (C), NIOPL (D), and NIOPGL (E). A: standard monosaccharides including Rha, Ara, Fuc, Xyl, Man, Glc, Gal; inositol used as internal reference.

MW values of the four NIOPs were determined as 780.90 (NIOPG), 1105.00 (NIOPF), 25.32 (NIOPL), and 10.28 kDa (NIOPGL), based on retention time relative to a series of standard glucans (Table 1). Monosaccharide compositions were determined by GC-MS, and profiles are shown in Fig. 4. Compositions of the four NIOPs were found to include Glc, Man, and Gal, Based on comparison with retention times of seven saccharide standards (inositol used as internal reference), we concluded that NIOPs were composed of Glc, Man, and Gal in respective molar ratios 92.17: 4.49: 3.35 (NIOPG), 68.73:18.33: 12.94 (NIOPF), 8.98: 52.36: 38.12 (NIOPL), and 26.43: 42.18:31.38 (NIOPGL) (Table 1).

NIOPs were subjected to periodate oxidation and Smith degradation, and glycosidic linkages were analyzed by GC-MS. Periodate consumption values for NIOPG, NIOPF, NIOPL, and NIOPGL were respectively 0.8462, 0.8787, 0.9159, and 0.9159 mol/mol Glc, indicating presence of 1→2, 1→4, and/or 1→6 linkages. Formic acid yields were >0 for each of the NIOPs, indicating presence of 1→6 linkage (Table 2). Smith degradation products were determined by GC-MS to be glycerol: Glc: erythritol in molar ratios 0.5107: 0.1142: 1 (NIOPG), 1.0078: 0.6420: 1(NIOPF), 25.1345: 16.9553: 1 (NIOPL), and 5.8569: 4.8408: 1 (NIOPGL). Proportions of 1→2, 1→3, 1→4, and 1→6 linkages were calculated, based on these values, as 3.52/ 32.39/ 47.08/ 17.01% (NIOPG), 3.05/ 38.45/ 32.17/ 26.33% (NIOPF), 49.64/ 27.04/ 4.69/ 18.63% (NIOPL), and 34.20/ 29.47/ 15.27/ 21.06% (NIOPGL) (Table 1). In conclusion, structural properties differed greatly among the four NIOPs, indicating a strong effect of carbon source on structures of *I. obliquus* mycelial polysaccharides.

### Effects of Carbon Source on Activities of *I. obliquus* Mycelial Polysaccharides

**Fig. 5:**
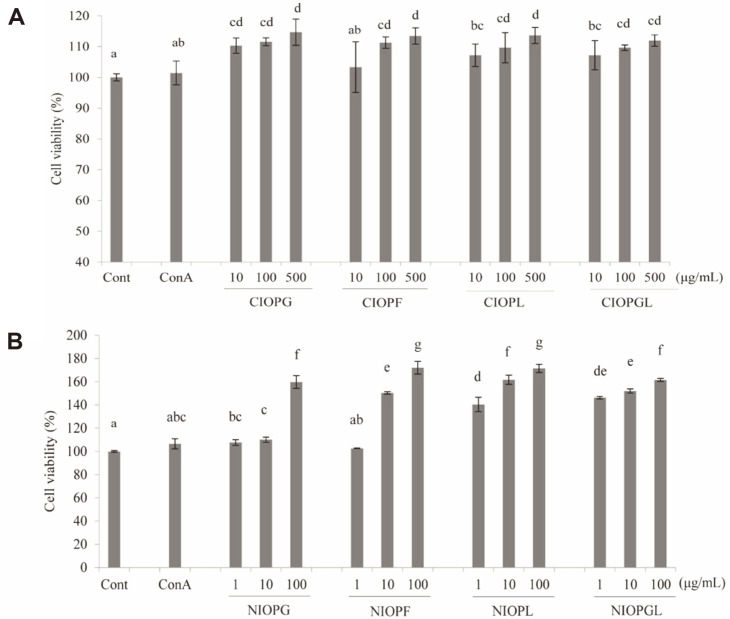
Effects of crude polysaccharides (A) and neutral polysaccharides (B) on proliferation of RAW264.7 cells in vitro. Cont: normal control group. ConA: positive control group, treated with 1 μg/ml. Differing letters above bars indicate significant (*p* < 0.05) differences according to Duncan’s multiple range test.

The effects of CIOPs and NIOPs on proliferation of macrophage cells (RAW264.7) were evaluated in vitro, using ConA (1 μg/ml) as positive control. The four CIOPs, at concentration 10-500 μg/ml, had a significant (*p* <0.05 or < 0.01), dose-dependent enhancing effect on proliferation in comparison with control (Fig. 5A). The same was true for the four NIOPs (Fig. 5B). Proliferation-enhancing effect in concentration range 1-100 μg/ml was stronger for NIOPs than for CIOPs, indicating that such activity was due to polysaccharides in CIOPs, not to other components. In this low concentration range, RAW264.7 cell viability was enhanced more strongly by NIOPL or NIOPGL than by NIOPG, NIOPF, or ConA (positive control) (Fig. 5B). These findings indicate that *I. obliquus* mycelial polysaccharides exert immune-enhancing activity, and that this activity is affected by carbon source used in fermentation.

**Fig. 6:**
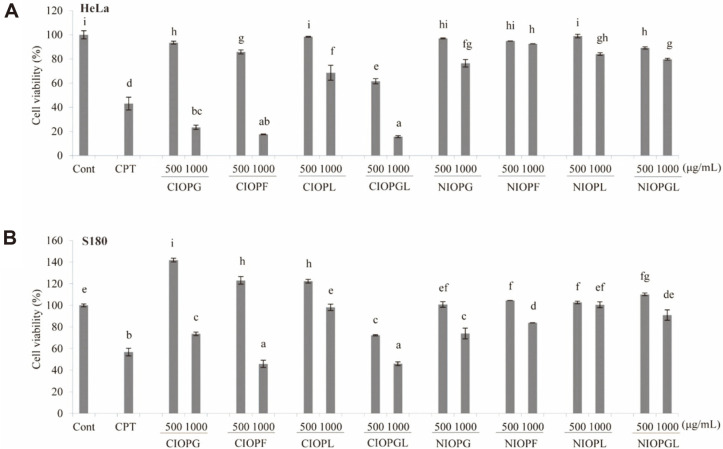
Effects of crude polysaccharides and neutral polysaccharides on proliferation of HeLa (A) and S180 (B) cells in vitro. Notations as in Fig. 5.

Anti-tumor activities of CIOPs and NIOPs were comparatively assessed based on effect on proliferation of HeLa and S180 cells in vitro, with CPT (600 μg/ml) as positive control. CIOPF and CIOPGL had significant (*p* < 0.01), dose-dependent proliferation-inhibitory effect on HeLa at all tested concentrations (Fig. 6A). At the highest concentration, proliferation-inhibitory effects of CIOPG, CIOPF, and CIOPGL on HeLa were stronger than that of CPT, and effects of CIOPs were stronger than those of NIOPs, suggesting that other components in CIOPs displayed anti-tumor activity. Proliferation-inhibitory effects of NIOPG and NIOPGL on HeLa were stronger than those of NIOPF and NIOPL, consistent with results for S180 cells (Fig. 6B). These findings indicate that *I. obliquus* mycelial polysaccharides display substantial anti-tumor activity, and that such activity is affected by carbon source.

### Effects of Carbon Source on Expression of Key Genes Involved in Mycelial Polysaccharide Biosynthetic Pathway

**Fig. 7:**
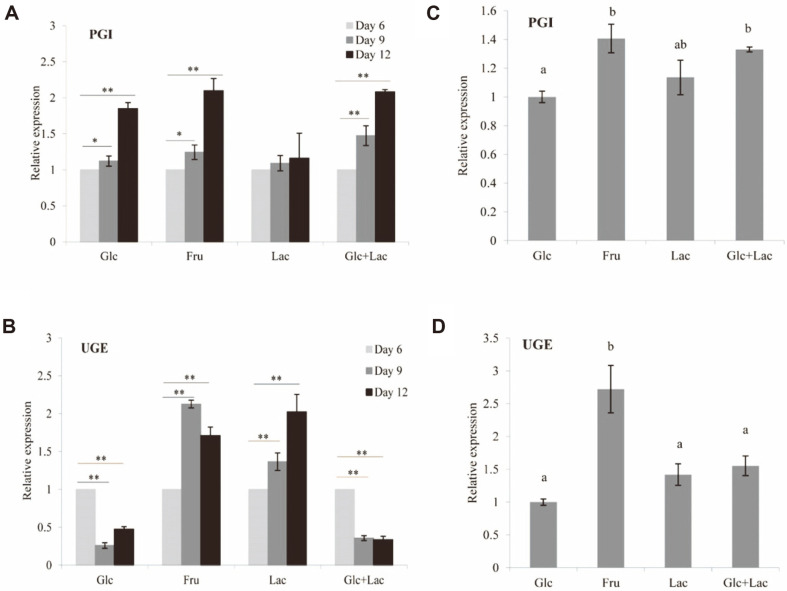
Effects of carbon source on transcription levels of genes encoding enzymes PGI (A) and UGE (B) involved in *I. obliquus* polysaccharide biosynthetic pathway. *p < 0.05, **p < 0.01 vs. Day 6. Differing letters above bars indicate significant (*p* < 0.05) differences according to Duncan’s multiple range test.

To evaluate the relationship between polysaccharide synthesis and carbon source used in fermentation, we analyzed expression levels of genes encoding PGI and UGE, two key enzymes involved in polysaccharide biosynthetic pathway. During a 12-d fermentation period, expression level of *pgi* in the four carbon source groups increased with time (Fig. 7A), consistent with the trend of increasing CIOP content. In the Glc, Fru, and Glc+Lac groups, this increase was significant. Therefore, PGI is evidently a key enzyme in regulation of polysaccharide synthesis, particularly for carbon sources Glc, Fru, or Glc+Lac. The order of *pgi* expression level at 12 days in the four groups was Fru > Glc+Lac > Lac> Glc (Fig. 7B), indicating a notable effect of carbon source on *pgi* expression.

In contrast, *uge* expression level did not vary notably regardless of whether the carbon source was Glc, Fru, or Glc+Lac (Fig. 7C). *uge* expression level showed significant time-dependent increase only for the Lac group, indicating that CIOPL synthesis is regulated mainly by UGE. *uge* expression level at the end of the 12-day period was significantly higher for Fru group than for the other three groups (Fig. 7D), indicating a notable effect of carbon source on *uge* expression.

## Discussion

Microbial polysaccharides (MPs) are secondary metabolites that display a variety of useful functions and have been widely applied in the food and pharmaceutical industries. Relationships among carbon sources, microbial biomass, and polysaccharide yield have long been a topic of great research interest for development and improvement of MP resources and related products [20-23]. In the present study, *I. obliquus* mycelial biomass and polysaccharide yield were investigated using different carbon sources. The results showed that these two indices were significantly higher for the Glc+Lac group (Glc and Lac used in combination as carbon source) than for other groups. Actually, nutritional carbon source requirements clearly differ for different medicinal fungi. For instance, in the research of mushroom *Phellinus linteus*, maximal mycelial growth and production of extracellular polysaccharides (EPs) were achieved when sorbitol was used as carbon source [24]. In the truffle *Tuber sinense*, use of Lac as carbon source promoted EP production, but not mycelial growth [20].

Artificial culture or fermentation techniques are often used for development of bioactive agents based on microbial compounds. In this context, structure and function of polysaccharides are more important than biomass and product yield. *Streptococcus pneumoniae*, when grown with Fru as carbon source, was unable to synthesize capsular polysaccharide, an important virulence factor [25]. *Bacillus subtilis*, a widely distributed and researched bacterium, when grown with burdock oligofructose as carbon source, produced a novel polysaccharide that displayed anti-cancer activity [26]. In this study, CIOPs and NIOPs generated by liquid fermentation using differing carbon sources, varied greatly in their immune-enhancing and anti-tumor activities. NIOPL and NIOPG displayed the strongest immune-enhancing and anti-tumor activities, respectively, suggesting potential application in drug or health food development.

Functions of MPs vary depending on structural differences (*e.g.*, monosaccharide composition, glycosidic linkages) which are affected by culture medium components, particularly carbon source. Peng *et al*. reported that mole percentages of four *Ganoderma lucidum* EPs (monosaccharides, Glc, Gal, Man) varied depending on mixed carbon source (Glc+Gal or Glc+Man, in molar ratio 1:1 or 1:2) [27]. Monosaccharide composition of *Nostoc flagelliforme* EPs similarly varied depending on carbon source condition (NaHCO_3_ in concentrations ranging from 0- 2.94 g/l) [28]. Kim, Lee and Yun observed that the composition of zooglan, an EP produced by the bacterium *Zoogloea ramigera*, varied depending on carbon source, with resulting changes in heavy metal adsorption characteristics [29]. In the present study, use of different carbon sources (Glc, Fru, Lac, Glc+Lac) greatly altered MW, monosaccharide composition, and glycosidic linkages of four NIOPs, resulting in varied immune-enhancing and anti-tumor activities.

**Fig. 8:**
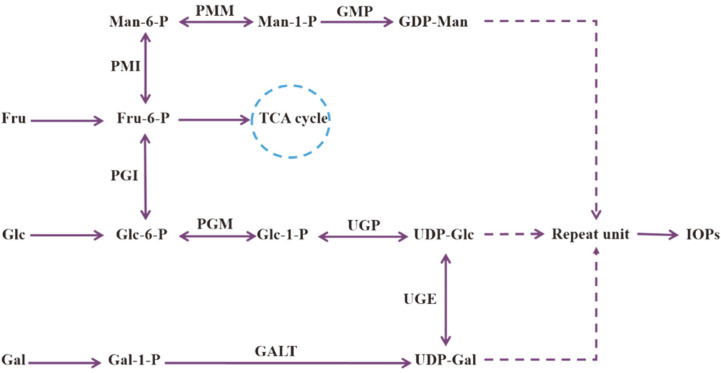
Proposed biosynthetic pathway for *I. obliquus* polysaccharides. PMM: phosphomannose mutase; GMP: GDP-Man pyrophosphorylase; PMI: phosphomannose isomerase; PGI: phosphoglucose isomerase; PGM: phosphoglucose mutase; UGP: UDP-Glc pyrophosphorylase; UGE: UDP-Gal-4-epimerase; GALT: galactose-1-phosphate uridylyltransferase.

Variations in structures and functions of MPs are generally associated with their biosynthetic pathways, in which relevant enzymes are up- or downregulated depending on carbon source. Enzymes involved in MP synthetic pathways clearly play key roles in production and structures of MPs [30]. In the case of *I. obliquus*, the IOP biosynthetic pathway, including a series of related enzymes (phosphomannose mutase [PMM], GDP-Man pyrophosphorylase [GMP], phosphomannose isomerase [PMI], PGI, UGE, and UDP-Glc pyrophosphorylase [UGP]), was deduced by monosaccharide compositions of IOPs and carbon sources used in this study (Fig. 8).

In this pathway, PGI represents an important control point and determines the conversion direction of Glc-6-phosphate (Glc-6-P), the precursor of sugar nucleotide UDP-Glc. Zhu *et al*. observed a strong correlation between PGI activity and production of intracellular polysaccharides in the medicinal fungus *Cordyceps militaris* [31]. PGI level may fluctuate under the fermentation condition using different sugars as carbon sources. In the research of *Ganoderma lucidum* strain 5.26, PGI level was lower when Suc (in comparison with Glc) was used as sole carbon source [32]. In this study, *pgi* gene expression under all four carbon source conditions was upregulated as fermentation time increased, consistent with the trend of IOP content. PGI is evidently a key enzyme in IOP synthetic pathway. *pgi* expression level was higher in Fru group than in other groups, and was correlated with high molar ratio of Glc in NIOPF monosaccharide composition. Under high-Fru condition, PGI catalyzes conversion of Fru-6-P to Glc-6-P. Similarly, NIOPL and NIOPGL contain high proportions of Man and its derivatives, as a result of high expression level of pgi, which catalyzes interconversion between Glc-6-P and Fru-6-P. Fru-6-P can be further converted to Man-6-P, Man-1-P, and consequent sugar nucleotide GDP-Man, by catalysis with related enzymes PMI, PMM, and GMP.

UGE level determines the direction of interconversion between UDP-Glc and UDP-Gal, which are precursors of polysaccharide repeat unit. Han *et al*. observed that UGE activity involved in *N. flagelliforme* EP synthesis was significantly inhibited when NaHCO_3_ at concentration 2.94 g/l was used as carbon source [28]. In the present study, UGE activity likewise varied depending on carbon source. *uge* expression level in Lac group was upregulated as fermentation time increased, indicating an important role of UGE in CIOPL synthesis. *uge* expression level in Glc, Fru, and Glc+Lac groups did not have similar effects, indicating that synthesis of corresponding IOPs may be associated with regulation of other key genes. Peng *et al*. reported correlation of higher Gal molar ratio in monosaccharide composition of *G. lucidum* EPs with higher PGM activity [33]. In *N. flagelliforme* EPs, higher proportion of Gal was associated with higher UDP-Glc dehydrogenase (UDPD) activity [28]. Relationships between carbon source and regulation of IOP synthesis will be clarified by future studies of other relevant enzymes in IOP biosynthetic pathways.

## Conclusion

Four crude polysaccharides (termed CIOPG, CIOPF, CIOPL, and CIOPGL) and four derived neutral polysaccharides (NIOPG, NIOPF, NIOPL, and NIOPGL) were extracted and purified from mycelia of *I. obliquus* generated by liquid fermentation using respective carbon sources Glc, Fru, Lac, and Glc+Lac. Structural analysis by FTIR, GC-MS, and several chemical methods revealed considerable variation in MW, monosaccharide composition, and glycosidic linkages of the four NIOPs. In vitro experiments showed that proliferation of macrophage cells (RAW264.7) and tumor cells (HeLa and S180) differed greatly among groups treated with the various CIOPs and NIOPs. qRT-PCR analysis revealed that expression levels of *pgi* and uge, two key genes involved in polysaccharide synthetic pathway, differed significantly depending on carbon source used. Carbon source clearly affected synthesis, structure, and function of fungal polysaccharides, based on differential regulation of expression levels of genes involved in polysaccharide biosynthesis.

## Figures and Tables

**Table 1:** Properties of crude polysaccharides and neutral polysaccharides from *I. obliquus* mycelia.

Polysaccharides	Carbohydrate (%)	Protein (%)	Molecular weight (kDa)	Monosaccharide composition (molar ratio)	Glycosidic linkages
CIOPG	33.48 ± 0.93	3.48 ± 0.74	-	-	-
CIOPF	18.88 ± 0.57	1.59 ± 0.57	-	-	-
CIOPL	20.77 ± 0.85	0.96 ± 1.13	-	-	-
CIOPGL	22.83 ± 1.44	1.01 ± 1.52	-	-	-
NIOPG	98.39 ± 1.83	0.23 ± 1.12	780.90	Glc, Man, Gal (92.17:4.49: 3.35)	1→2, 1→3, 1→4, 1→6 (3.52%/ 32.39%/ 47.08%/ 17.01%)
NIOPF	96.21 ± 1.60	0.83 ± 0.07	1105.00	Glc, Man, Gal (68.73: 18.33: 12.94)	1→2, 1→3, 1→4, 1→6 (3.05%/ 38.45%/ 32.17%/2 6.33%)
NIOPL	90.60 ± 2.28	0.13 ± 0.29	25.32	Glc, Man, Gal (8.98: 52.36: 38.12)	1→2, 1→3, 1→4, 1→6 (49.64%/ 27.04%/ 4.69%/ 18.63%)
NIOPGL	90.96 ± 1.76	0.35 ± 0.22	10.28	Glc, Man, Gal (26.43:42.18: 31.38)	1→2, 1→3, 1→4, 1→6 (34.20%/ 29.47%/ 15.27%/ 21.06%)

**Table 2:** Results of periodate oxidation/Smith degradation of *Inonotus obliquus* neutral polysaccharides.

Linkage	Periodate consumption (mol/mol Glc)	Formic acid production (mol/mol Glc)	Smith degradation products
1→2	1	nd^a^	Glycerol
1→3	nd	nd	Glucose/mannose
1→4	1	nd	Erythritol
1→6	2	1	Glycerol
NIOPG	0.8462	0.1701	Glycerol: Glucose: Erythritol= 0.5107:0.1142:1
NIOPF	0.8787	0.2632	Glycerol: Glucose: Erythritol= 1.0078:0.6420:1
NIOPL	0.9159	0.1863	Glycerol: Glucose: Erythritol=25.1345:16.9553:1
NIOPGL	0.9159	0.2106	Glycerol: Glucose: Erythritol=5.8569: 4.8408:1

^a^not detected.
